# Assessment of the Geochemical Availability and Ecological Risk of Trace Elements in Marine Sediments of the Tremiti Islands

**DOI:** 10.3390/molecules30204051

**Published:** 2025-10-11

**Authors:** Martina Fattobene, Raffaele Emanuele Russo, Mario Berrettoni, María Dolores Galindo-Riaño

**Affiliations:** 1 School of Science and Technology, Chemistry Division, University of Camerino, Via Madonna delle Carceri—ChIP, 62032 Camerino, Italy; martina.fattobene@unicam.it (M.F.); raffaele.russo@unicam.it (R.E.R.); 2 Department of Analytical Chemistry, Institute of Biomolecules (INBIO), Faculty of Sciences, CEI-MAR, Campus Río San Pedro, University of Cádiz, 11510 Puerto Real, Spain

**Keywords:** ecological risk assessment, BCR, environmental monitoring, marine sediments, geochemical availability

## Abstract

Marine protected areas (MPAs) near the coast are a global concern due to potential impact of anthropogenic activities highly relevant when it comes to trace elements pollution in sediment. This study aims to assess the levels of trace elements in sediment, their potential mobility and the ecological risk in Tremiti Islands, a sensitive and vulnerable MPA. Sediment was analyzed for granulometry, mineralogy, pseudo-metal concentrations and available fractions using BCR method. Statistical analysis and different pollution and ecological risk indices were applied to interpret the results, determine the contamination levels and assess the element availability and their potential impact using Sediment Quality Guidelines. Spatial variability in grain size and mineralogy was found across the sampling sites. The finer quartz-rich sediments exhibiting higher trace element concentrations. Site-specific enrichments were evident for As and Zn at Cala Spido and for Pb at Cala Matano. Cu and Mn showed notable potential bioavailability with residual fractions below 30% at all sites; low Cd concentrations were found, but it was highly available. Cala Spido and Grotta del Sale showed higher contamination-degree, while Pagliai and Cala Matano stood out for their higher ecological risk and availability indexes. These findings demonstrated that even within a Marine Protected Area, site-specific anthropogenic pressures can significantly influence sediment quality and ecological risk.

## 1. Introduction

Marine sediments function both as sinks and potential secondary sources of trace elements (TEs: heavy metals and metalloids), depending on their physicochemical characteristics, which influence element mobility and availability. It is estimated that >99% of TEs entering an aquatic ecosystem accumulate in surface sediments, which poses a risk due to their significant potential toxicity [1]. Chemical pollution is considered a threat to marine protected areas (MPAs), and the scientific community is encouraged to study pollution in MPAs worldwide including its sources, extent and effects on ecosystems and the biota [2].

In the review by Abessa et al. on the global pollution status of marine protected areas, the Tremiti Islands were described as an area of interest but with limited studies regarding its environmental quality [2]. These islands are located in the Adriatic Sea, which is considered a semi-enclosed sea with weak circulation and favoring sedimentation and metal retention [3]. The Tremiti Islands Marine Protected Area (MPA), identified according to the low n.979/1982 and established by the decree-law 14 July 1989, represents a vulnerable zone subject to natural and anthropogenic pressures. Potential anthropogenic sources include boat traffic, port activities and seasonal tourism, which may locally enhance trace elements inputs. Also, limited water circulation may favor contaminant accumulation. These islands were classified by the Abessa study as showing initial evidence of partial contamination by iron and other contaminants, such as tributyltin, with deleterious effects on some organisms [2]. Given the importance of these areas, the study emphasized the need for the ongoing monitoring of pollution and research to better understand the impact of chemical pollution on MPAs globally. Some subsequent studies were conducted on these islands identifying them as a critical point of metal contamination in the coastal of the Adriatic Sea, given the high concentrations of V, Cd and Pb found in mussel tissues. The presence of V suggested the potential impact from oil and gas platforms or maritime traffic [4]. High levels of heavy metals including Co, Ni, Hg, Cr and Pb were detected in mesozooplankton [5]. Previous research conducted by the authors of this paper focused on the possible stress factors causing the growing regression of *Posidonia oceanica* on these islands. The study reported bioconcentration factors (BCFs) greater than one for almost all analyzed TEs (Ag, Ba, Cd, Cr, Cu, Mn, Mo, Ni, Pb, Sb, Se, Sn, Zn), with very high values for Sn, Mo, Zn and Ni. The sediments were extremely enriched in Ba and Ti at all sampling points, and in Cd and Pb at Pagliai. The levels of metal concentration in the outer leaves of Posidonia were influenced by the amount of TE present in the sediment at Pagliai site [6]. Therefore, despite its designation as a protected area, comprehensive studies are necessary to characterize the spatial and temporal variability of TEs contamination in sediments of Tremiti Islands due to the lack of data.

Grain size distribution is a key parameter in the characterization of metal and metalloid pollution in sediments. Finer particles, such as silts and clays, offer larger surface areas and higher cation exchange capacities, enhancing the adsorption of TEs and organic contaminants [7]. Finer sediments are generally associated with higher contamination levels due to their capacity to retain TEs, particularly in bioavailable and moderately available phases [8]. The mineralogical composition of sediment is another important factor that determines their mobility and availability. Different mineral compositions have different affinities for metals. Understanding heavy metal contamination in sediments and establishing the natural geochemical background of metals is crucially important. Ignoring local mineralogical characteristics can lead to incorrect assessment, confusing natural (lithogenic) enrichments with anthropogenic contamination [9].

The concentration of TEs in marine sediments is misleading for assessing ecological risk, being essential for knowing their potential availability. The European Communities Bureau of Reference (BCR) modified extraction method is commonly used to differentiate the available fractions (easily extractable, reducible and oxidizable) from residual phase.

This protocol is a fundamental technique for environmental scientists to move beyond simple total metal measurements and gain a deeper understanding of the ecological impact of heavy metal pollution in marine ecosystems, offering insight into mobility and potential geochemical and biological availability [10].

The interpretation of geochemical and ecological risk studies of metal pollution in sediment is widely carried out by using index-based approaches [11,12]. While indices such as *Cf* (Contamination factor), *Igeo* (Geoaccumulation index) and *EF* (Enrichment factor) identify pollution hotspots and normalize metal concentrations for enhanced spatial or temporal interpretation, integrative indices like the Contamination Degree (*C_d_*), Potential Ecological Risk Index (*PERI*), and Availability Index (*AI*) provide insights into the ecological significance of contamination, identifying which trace elements are likely to affect benthic organisms and seagrass meadows [1,13]. In this sense, the application of *PERI* allows for risk-oriented interpretation by integrating contamination levels with toxicity response coefficients. However, their accuracy and utility are critically dependent on the availability and precision of background or reference values, the existence of established toxicity coefficients for all contaminants, and the necessity to integrate these results with other environmental data for a comprehensive ecological understanding [13].

In this context, the proposed research approach involved granulometric and mineralogical analysis, as well as combining the pseudo-total (aqua regia extractable) and fractionated element analyses through the BCR protocol in sediment from Tremiti Island. The application of multivariate statistical tools (Principal Component Analysis and Pearson correlations) and different pollution and ecological risk indices (Contamination indices (*Cf*, *C_d_* and *mC_d_*), Enrichment factor (*EF*), Geoaccumulation Index (*Igeo*), Potential Ecological Risk Index (*PERI*) and Availability Index (*AI*)) were also proposed to interpret the results. This study aimed to determine the levels of sediment contamination in this sensitive Marine Protected Area (MPA), explore the relationships between TEs and sediment characteristics, assess the metal availability and evaluate the potential ecological risk of TEs to the environment and the biota. In this way, the severity and ecological implications of trace element contamination and the effect on biota may be established.

Previous research on sediment contamination in the Adriatic Sea has mainly addressed estuarine or industrialized areas [14] and trace metal distribution in coastal sediment from the Adriatic Sea. In contrast, the Tremiti Islands provide an opportunity to explore contamination dynamics in a relatively isolated but heavily visited MPA, where natural drivers (e.g., lithogenic inputs) and anthropogenic pressures (e.g., port activities, seasonal tourism) coexist. This integrative approach moves beyond simple concentration reporting, offering a risk-based environmental assessment that directly links sediment chemistry to potential ecological impacts on *Posidonia oceanica* meadows. In this work, a new sediment sampling campaign was conducted in January 2025, and the obtained trace element data were analyzed. The results were then compared with those from two previous campaigns carried out in July and September 2023, which were used for temporal comparison.

## 2. Results and Discussion

### 2.1. Granulometric Composition, Spatial-Bathymetric Trends and XRD Analysis

**Figure 1:**
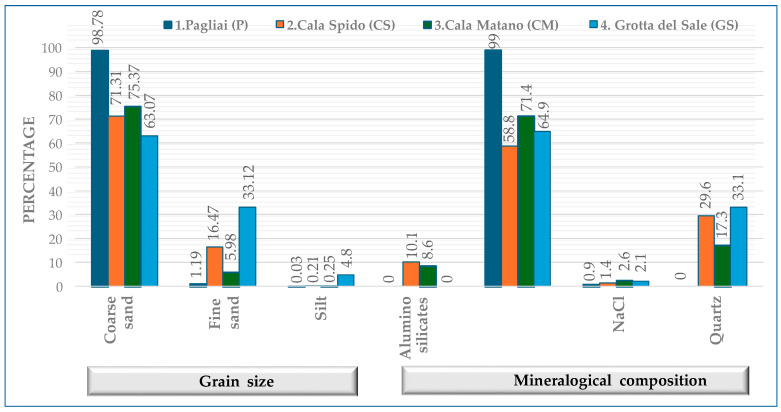
Percentage of grain size and mineralogical composition of sediments. Aluminosilicates included the following: Albite (Ca-bearing) and Albite (Na-bearing); Carbonates included the following: Aragonite CaCO_3_, Calcite CaCO_3_, Calcite (Mg-bearing), Dolomite CaMg(CO_3_)_2_ and Dolomite Ca(Mg,Fe)(CO_3_)_2_.

Figure 1 shows that along the San Domino coast, there is a variation in sediment grain size and mineral content. At P (10.3 m), sediments are dominated by coarse sand and dolomite, characteristic of a high-energy environment. Moving to CS (10.0 m), fine sand increases, and the mineral composition changes with the appearance of quartz, calcite and Fe-bearing dolomite. This suggests a shift toward a moderately lower-energy environment with more varied sediment sources. At CM (15.0 m), the mineral assemblage includes aragonitic calcite, aragonite and Fe-bearing dolomite, indicating a combination of biogenic and terrigenous inputs. Aragonite, less stable than calcite, can arise from marine organisms such as corals and shell-producing fauna. Its presence, along with aragonitic calcite, points to active biogenic contributions, while its eventual transformation into calcite reflects ongoing diagenetic processes. Finally, GS (19.0 m) contains the finest sediments and the most varied mineralogy, including quartz, calcite and dolomite. Calcite, formed via biochemical processes in sedimentary environments and the most abundant mineral in the dataset, is consistently associated with finer grain sizes across the sites, supporting the observed trend of increased mineralogical complexity and carbonate accumulation in lower-energy zones.

Sediment granulometry influenced metal distribution: sites dominated by finer fractions (CS, GS) exhibited higher trace metal concentrations, consistent with their higher adsorption capacity. XRD results indicated a mineralogical shift, with quartz enrichment in fine sediments associated with elevated Cu, Zn and Pb, supporting the role of granulometry and mineralogy as primary controls.

Granulometric and mineralogical analyses were performed only in the framework of the present study; thus, no temporal variability could be assessed. Results highlight spatial differences among sites.

Mineralogical analyses revealed carbonate enrichment at GS, consistent with shallow environments influenced by seagrass meadows, whereas quartz and feldspar dominated sediments at CS and P, reflecting higher terrigenous inputs. These patterns explain the spatial heterogeneity in contamination indices, as carbonate-rich sediments tend to dilute trace metals, while quartz-dominated sediments concentrate them in finer fractions. A study in the Gulf of Oristano (Sardinia) found that *P. oceanica* meadows promoted the accumulation of biogenic carbonate sediments in certain areas protected from waves [15]. However, dolomite is mainly generated in situ after diagenesis processes, rather than having been transported and deposited.

The association between sediment grain size/mineralogy and TE content was further supported by statistical analysis. Sites with finer sediments and quartz enrichment (CS, GS) exhibited higher concentrations of Cu, Zn and Pb, while carbonate-rich coarser sediments (P) displayed lower associations, indicating the primary role of grain size and quartz-rich fine fractions in TE retention.

### 2.2. Trace Element Content and Contamination Assessment

The results (Table 1) show a wide variation in average metal concentrations between sites. For instance, Al ranges from 457.69 ppm at P to over 5210 ppm at GS, suggesting different environmental conditions or contamination sources. High levels of Al and Fe may reflect local geology or anthropogenic influences. Comparison of pseudo-total concentrations with BCR fractionation (Table 1) confirmed that Cr and Ni, although abundant, were mostly confined to the residual fraction, indicating that they are strongly bound to stable lithogenic components such as silicates and crystalline oxides. This association greatly limits their geochemical mobility and ecological relevance. Conversely, Cu and Zn showed a relevant proportion in acid-soluble and reducible fractions, suggesting greater potential mobility. Pb was distributed between reducible and residual phases, whereas Cd, despite its low total levels, was largely associated with the acid-soluble fraction, making it the most potentially bioavailable and ecologically concerning element.

By merging this data with the 2023 dataset, it is possible to observe the patterns highlighted by the PCA. This tool allows us to know the overall structure of the data, identifying which variables (loadings) and samples (scores) are similar or different from each other. The loading and score plots display groups of similar data points and show which variables characterize each group.

**Figure 2:**
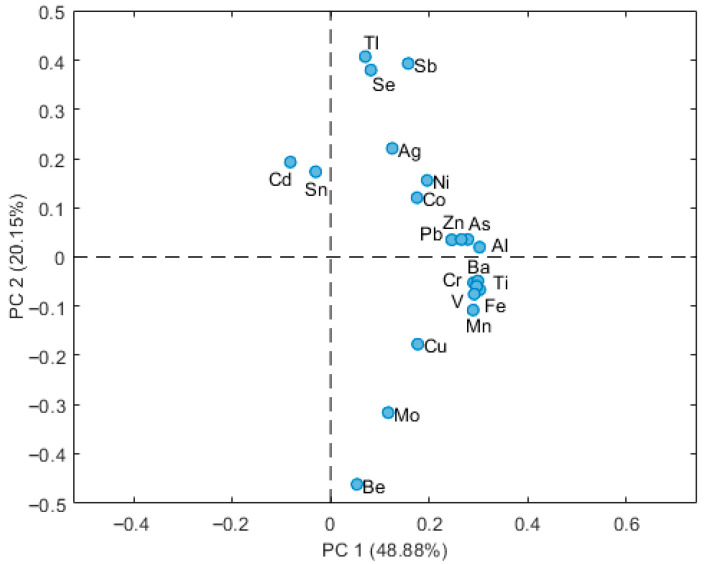
Loading plot of the first two principal components (PC1 and PC2) obtained from pseudo-total TEs content. Data from the whole available dataset (July 2023, September 2023, January 2025).

**Figure 3:**
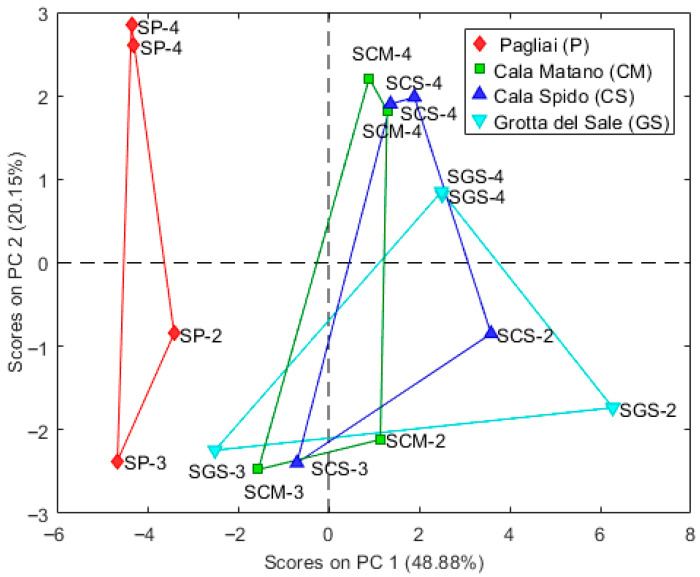
Score plot of the first two PCs showing the distribution based on the sampling sites. Data from pseudo-total TE content and whole dataset (July 2023, September 2023, January 2025).

The loading plot (Figure 2) shows the distribution of the TEs along the first two principal components, PC1 (48.88%) and PC2 (20.15%), which together explain approximately 69% of the total variance. The most important component (PC1) expresses the overall content of elements, and it is correlated with TEs with higher concentrations in the sediment. The higher the concentration of the elements, the stronger the positive correlation with PC1. Conversely, TEs with low concentrations correlated more with PC2, both positively and negatively. From the score plot (Figure 3), we note that the samples from P are clearly separated along PC1, staying on the left, indicating lower element contents at this site compared to the other locations (scores on PC1: from −4.8 to 6.4). In contrast, samples from CM, CS and GS show partial overlap but still exhibit separation with this order for the PC1 scores: SG > CS > CM. The different correlations of sampling sites with PC2 had lower scores (from −2.4 to 3), principally for those elements with low concentrations. These results suggest site-specific differences as well as seasonal variation in TE content, depending also on the level of TE in the sediment. Thus the loading plot (Figure 2) provides insight into which elements contribute most to the observed separation. The differentiation between sites along PC1 appears to be driven by increasing concentrations of nearly all TEs. Conversely, PC2 contrasts the group of elements Tl, Se, Sb, Ag and Cd against the group Be, Mo and Cu. Combining the loading plot with the score plot along PC2, it appears clearly that samples 2025 show higher values of the first group, and lower values of the second one, with respect to the 2023 samples. Overall, the PCA highlights clear compositional differences between sampling sites, with P samples that are consistently positioned on the negative side of PC1 (low values of almost all the elements), possibly linked to localized environmental or geological factors.

BCR results indicate that Cu, Pb and Mn were the most bioavailable elements, with residual fractions below 30% at CS and CM. These sites also correspond to finer sediments, confirming the role of grain size in enhancing TE mobility. Conversely, Zn, Fe and Al were mainly residual (>50–90%), suggesting limited bioavailability. From an ecological perspective, the bioavailable fraction of Cu and Pb may induce oxidative stress and growth inhibition in benthic fauna, while Mn, although essential, may become toxic at elevated concentrations [16,17]. To further explore the temporal variability of metal concentrations of samples from July 2023 (SCS-2, SGS-2, SP-2, SCM-2), September 2023 (SCS-3,SGS-3,P-3,CM-3) and January 2025 (SCS-4, SGS-4, SP-4, SCM-4) and their associations with sediment characteristics, PCA-score plot and correlation analyses were applied.

From a contamination-degree perspective, GS and CS show the highest cumulative contamination (*C_d_* and *mC_d_*), with GS also exhibiting the highest PLI. However, considering ecological risk and mobility, *PERI* peaks at P and CS, whereas GS displays the lowest *PERI* and lowest *AI_tot_*, indicating reduced overall availability. Site-specific enrichments are evident for As and Zn at CS (highest *EF*), and for Pb at CM.

**Figure 4:**
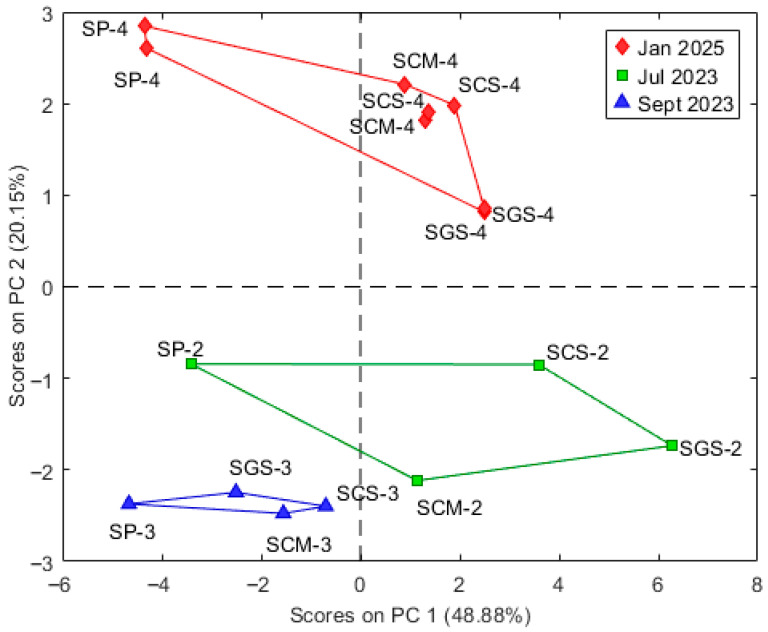
Score plot showing data grouped according to the sampling date, where the numbers -2, -3 and -4 indicate the sampling time July 2023, September 2023 and January 2025, respectively.

The PCA score plot in Figure 4 reports data grouped according to the sampling date. It reveals a clear temporal separation of the samples collected in 2023 and 2025, particularly along PC2 (20.15%). Samples from 2025 (red) are distinctly shifted from those collected in July and September 2023 (green and blue), indicating significant changes in elemental composition over time. The loading plot (Figure 2) shows that this temporal differentiation is primarily driven by increased levels of metals with positive loadings on PC2.

**Figure 5:**
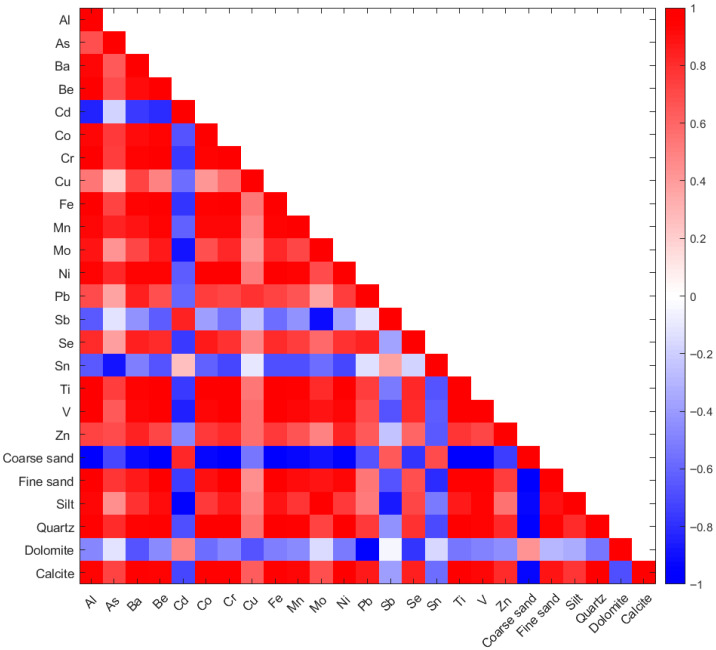
Pearson correlation matrix among metal concentrations, most abundant mineralogical fractions (quartz, dolomite, calcite) and granulometric components (coarse sand, fine sand, silt).

The Pearson correlation matrix (Figure 5) provides valuable insights into the relationships among metal concentrations, mineralogical composition (quartz, dolomite, calcite) and grain size fractions (coarse sand, fine sand and silt). These correlations allow for a better understanding of the mechanisms influencing metal distribution and potential sources, whether lithogenic or anthropogenic. The Pearson correlation matrix (Figure 5) shows that positive associations between trace metals and sediment parameters were primarily linked to the fine-grained fractions (silt and fine sand) and to quartz and calcite. In particular, Cu, Zn and Pb co-varied more consistently than other metals, which varied more across sites and were generally weaker. Conversely, dolomite showed predominantly negative or weak correlations with trace metals, suggesting a more limited role in metal binding. The negative association of TEs with coarser grain sizes (particularly coarse sand) and dolomite, supports the idea that diagenetic carbonate-rich (dolomite) and coarser fractions are less involved in the retention or accumulation of metals. In contrast, calcite had a behavior quite similar to quartz. Moreover, metals such as Fe and Al exhibited strong mutual correlation (r ≈ 0.96), which aligns with their common lithogenic origin. The high correlations between fine sand/silt and several metals (e.g., Cu, Zn, Pb) emphasize the importance of grain size in controlling metal distribution. The combined evidence from PCA and Pearson correlation supports an interpretation where textural sorting and fine mineralogy explain most of the observed spatial variability in trace metal loads.

Grain size controls not only the distribution but also the transformation of trace metals. Fine-grained sediments enhance sorption and co-precipitation with Fe–Mn oxides and organic matter. Under diagenetic reduction, these carriers dissolve, remobilizing metals into pore waters [18]. Sulfide precipitation may subsequently transform them into more stable, less bioavailable phases (e.g., PbS, CuS). In our study, Cu and Mn exhibited low %F4 at CS and GS, suggesting that fine sediments at these sites may act as both reservoirs and potential sources of mobilizable metals, depending on redox conditions [8].

All the metals’ concentrations were compared with the most empirical SQGs (Threshold-Effects Level (TEL)/Possible-Effects Level and Effects Range Low (ERL)/Effects Range Median (ERM) [19,20]) and none of the values exceeded the limits. The other calculated indexes are reported in Table 2.

The values of background levels reported by [9,21] were used because of the proximity of sampling sites. Be, Mo, Sb, Se, Sn and Tl were not considered because of the absence of reference values for the background level. Fe and Al were also not considered because of natural high content.

The analysis of TEs revealed moderate and localized contamination, with notable spatial differences. Overall, GS is the most contaminated in terms of cumulative indices (*C_d_*, *mC_d_*, *PLI*), but this does not translate into the highest ecological risk due to its low *PERI* and low *AI_tot_*. CS combines elevated contamination degree with high *PERI* and strong enrichments for As and Zn, thus representing a priority hotspot. Conversely, P shows minimal cumulative contamination yet the highest *PERI* and *AI_tot_*. Although limited, comparison of 2023 and 2025 data suggest a reduction in *C_d_* and *PERI* values, but no temporal assessment of granulometry/mineralogy was performed. Global indices like *mC_d_* and *PLI* support the overall assessment of low contamination (*PLI* < 1), although a gradual increase is observed from north to south. The *PERI* remains low at all sites and *AI_tot_* shows a decreasing trend in the following order: P > CM > CS > GS.

**Figure 6:**
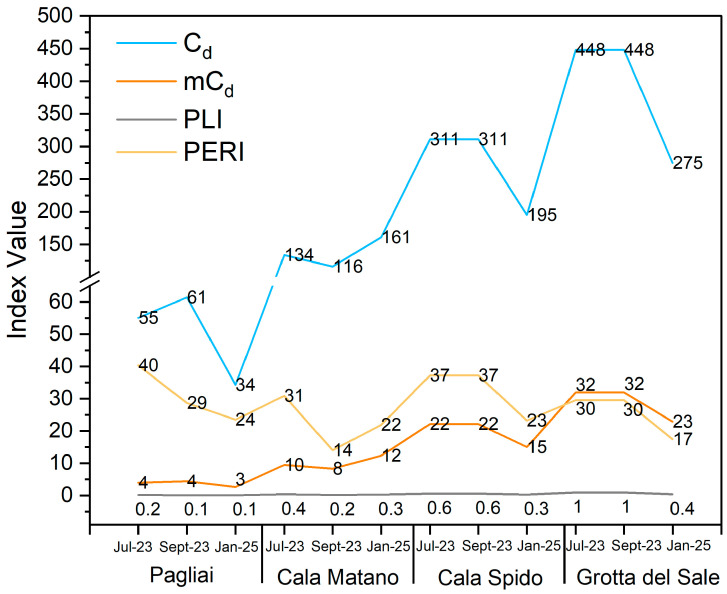
Temporal variation in pollution indexes (*C_d_*, *mC_d_*, *PLI* and *PERI*) across four sampling sites over three sampling times (July 23, September 23 and January 25).

The temporal trends across sites reveal differing contamination patterns as shown in Figure 6. P shows a peak in *C_d_* and *mC_d_* levels in September 2023, followed by a decline by January 2025, alongside a steady decrease in the *PERI*. At CM, the data show more fluctuation, with a decrease followed by a rise in January 2025, possibly indicating contamination inputs. CS exhibits stable *C_d_* and *mC_d_* values during the first two samplings and reductions in *PLI* and *PERI*. GS reports the highest overall contamination levels in the first two periods but also shows improvement by January 2025. Among all sites, GS and CS emerge as the most impacted from an ecotoxicological perspective.

**Figure 7:**
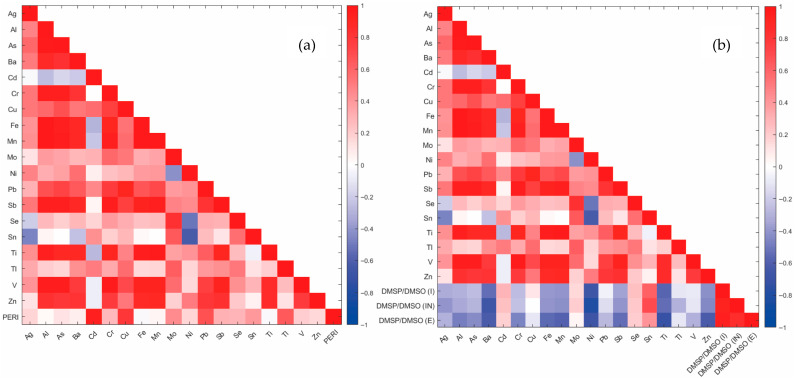
Correlation matrices for (**a**) the *PERI*; (**b**) DMSP/DMSO ratio in leaves of *P. oceanica*. (Biological data are from 2023 campaigns and are included here for comparison. No plant material was analyzed in the present study).

Figure 7 shows the correlation matrices of trace element concentrations (from 2023) for two ecological factors (from 2023): the *PERI* and the DMSP/DMSO ratio in leaves of *P. oceanica*, providing insight into the behavior and potential impact of metals in *Posidonia oceanica* meadows, which are regressing. The DMSP/DMSO ratio is defined as the ratio of dimethyl sulfoniopropionate (DMSP) to dimethyl sulfoxide (DMSO) concentrations. This ratio is used as an indicator to evaluate oxidative stress levels in the plant, as naturally present DMSP is oxidized to DMSO under stress, causing the ratio to decrease. A higher DMSP/DMSO ratio indicates lower oxidative stress [6]. The correlation matrix a shows a positive correlation between metal in sediment and *PERI*, which is logical since the higher the metal concentration, the higher the *PERI*; e.g., Cd, Cu, Mo, Pb, Sn and Tl show a medium–strong positive correlation. The correlation matrices for *Posidonia oceanica* (Figure 7b) derive from previously published data [6] and are reported solely to provide ecological context. No direct measurements on seagrass tissues were conducted in this work. The matrix b shows a general negative correlation between TE content and DMSP/DMSO ratio in every type of *P. oceanica* leaf, which is correct since a low ratio indicates a high oxidative stress of the plant except for Cd, Mo, Se and Sn, which show a positive but not significant correlation since the DMSP/DMSO ratio may depend on different environmental factors. This pattern suggests that, even in the absence of updated biotic data, metals may be related to the health status of the plant.

The observed decrease in *C_d_* and associated indices between 2023 [6] and 2025 should therefore be regarded as preliminary. Additional campaigns are required to confirm whether this reflects reduced anthropogenic input or natural variability.

## 3. Materials and Methods

### 3.1. Sampling Area

**Figure 8:**
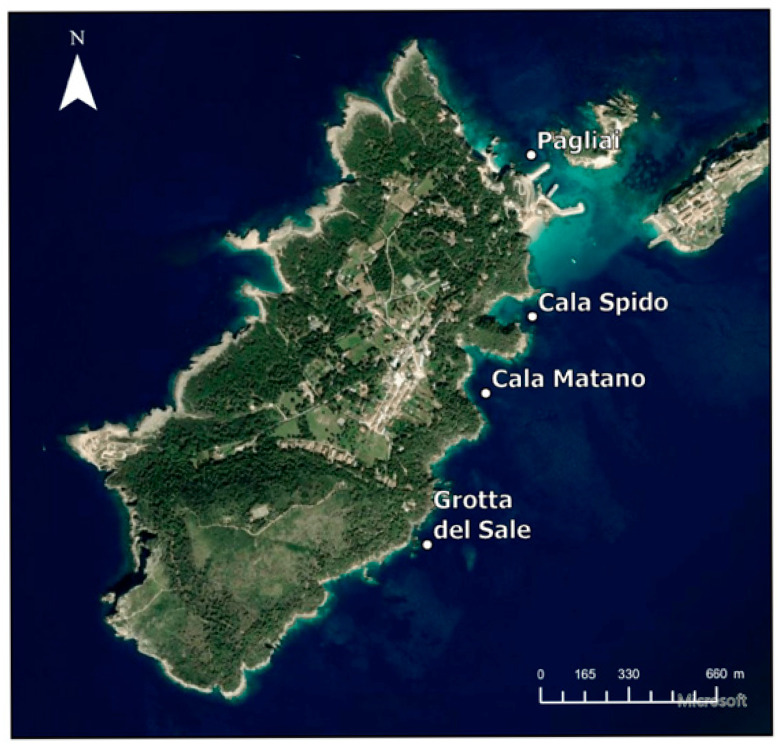
San Domino Island view with sampling sites. Map elaborated by the authors using ArcGIS Pro 3.3 (licensed academic version).

The Tremiti Islands constitute a small archipelago located in Italy (Puglia Region, Adriatic Sea). A targeted sampling campaign was conducted along the coastal marine environment of San Domino Island. The sampling sites were the same as in previous study achieved by the authors in 2023 [6], following the detection of TE accumulation in samples [6] as well as significant annual changes in the density and health of *Posidonia oceanica* meadows [21]. The designated sites—Cala Pagliai (P), Cala Matano (CM), Cala Spido (CS), and Grotta del Sale (GS) (Table 3)—are all located within the southern side of the island and are relatively close to one another (300–600 m) (Figure 8). The area features shallow sandy bottoms that support the growth and stability of *Posidonia oceanica* meadows and is geologically composed of layered carbonate rocks shaped by strong karstic, marine and gravitational forces. On San Domino, karst landforms such as circular depressions and sinkholes are prominently developed, frequently incised into calcrete surfaces and infilled with wind-blown sands [22,23]. The region is subject to elevated levels of marine traffic, primarily due to transportation routes and intense seasonal tourism. Sediment samples were obtained near the *P. oceanica* bundles by scuba divers in January 2025 during the morning under calm sea conditions (<0.5 m waves) without atmospheric precipitation to avoid any type of contamination. Surface sediment (0–5 cm) was collected using 1000 mL PTFE bottles at the designated points. After collection, the bottles were sealed and stored at 4 °C to maintain sample integrity. The samples were sieved with a 2 mm sieve before the analysis. X-Ray diffraction in the sample (<2 mm) grinded with an agate mortar was performed to detect the most abundant mineralogical fractions for each sampling site.

### 3.2. Granulometric and Mineralogical Analysis

Grain size analysis was performed by sieving the sediment through a series of stacked sieves, progressively separating the sample into smaller size fractions by using a vibrating sieve shaker [24]. The material collected was dried at 40 °C and weighed to determine its proportion relative to the entire sample [25]. Between 200 and 300 g of sediment were weighed and then sieved with 3 sieves used to categorize sediments into coarse (>0.250 mm), medium (0.250–0.063 mm) and fine fractions (<0.063 mm). The sieving process was carried out with an FTS-0200 model IRIS electromagnetic sieve shaker from Filtra Vibration S.L. (Barcelona, Spain), for 4 h at power 6 and cycle 3. At the end, each fraction of sample was weighed, packed in polyethylene (LDPE) bottles and stored at room temperature until analysis.

Mineralogical composition was determined by X-ray diffraction using a Bruker D8 Advance diffractometer (Bruker AXS GmbH, Karlsruhe, Germany) operating at 40 kV, 40 mA with Cu Kα radiation.

### 3.3. Trace Element Analysis

Pseudo-total fraction elements of Ag, Al, As, Ba, Be, Cd, Co, Cr, Cu, Fe, Mn, Mo, Ni, Pb, Sb, Se, Sn, Ti, Tl, V and Zn were analyzed by weighing approximately 0.5 g of sediment into a PTFE microwave digestion vessel through 3 mL of HCl 36% *w*/*w* from Supelco and 9 mL of HNO_3_ 65% *w*/*w* from PanReac AppliChem (Darmstadt, Germany). The digestion was accomplished using a Milestone Ethos One High Performance Microwave Digestion System (Milestone Srl, Bergamo, Italy) at 230 °C, increasing 8 °C/min for 35 min, following EPA Method 3051A [26]. The solutions were filtered through a 0.45 µm regenerate cellulose membrane filter brought to a total volume of 25 mL and analyzed by ICP-OES. Extraction of the available fraction was performed using the BCR protocol [27]. The BCR protocol was selected as it is standardized by the European Community Bureau of Reference and provides more reproducible and ecologically relevant information compared to other protocols such as Tessier’s fractionation [10,28]. The quantitative determination of pseudo-total metals and metals in BCR-residue was performed using Spectrogreen ICP-OES from SpectroAmetek (Karnataka, India). The quantification was carried out using external standards calibration curve of the MultiElement Standards (504311, Carlo Erba Reagents srl, Milan, Italy) and individual standards for Al (61935, Sigma Aldrich, St. Louis, MO, USA), Fe (119761, Merck, Darmstadt, Germany) and Sn (43922907, Merck, Darmstadt, Germany).

The three-step BCR (Community Bureau of Reference) sequential extraction procedure, developed by the Community Bureau of Reference (now the Standards, Measurements, and Testing Program), is a chemical and ecotoxicological analysis used to identify the chemical forms of TEs in samples (from phase F1 to F3). After completing the sequential extraction phases, the residual TEs content is determined through an additional phase (F4).

The original BCR procedure involves sequential extraction using the following:

- Phase 1: 0.11 mol L^−1^ acetic acid for soluble, exchangeable and carbonate-bound TEs.
- Phase 2: 0.5 mol L^−1^ hydroxylammonium chloride at pH 1.5 for elements occluded in easily reducible iron and manganese oxides.
- Phase 3: 8.8 mol L^−1^ hydrogen peroxide followed by 1 mol L^−1^ ammonium acetate at pH 2 for TEs bound to organic matter and sulfides.
- Phase 4: The residue from Phase 3 is digested with aqua regia for TEs fixed in the crystalline lattice of primary and secondary minerals and the sum of TEs content extracted sequentially (Σ Phase 1 + Phase 2 + Phase 3 + Phase 4) is compared to the TEs content extracted in a single step with aqua regia (defined here as pseudo-total content; aqua regia extractable in the previous work).

It is possible to refer to the sequentially extracted fractions as “easily extractable”, “reducible”, “oxidizable” and “residual” metals, respectively.

Quality control was ensured using multi-element standard calibration curves and internal consistency checks. Agreement between pseudo-total concentrations and the sum of BCR fractions was within 10%, indicating acceptable reliability of the sequential extraction (acceptable value for TEs recoveries > 85%) [10]; in the same way the BCR-701 reference material (lake sediment) was used to satisfactorily validate the results of the pseudo-total content and F4 fraction analyses (Appendix A). All the reagents and solvents were of analytical or Suprapur grade, and all the solutions were prepared using ultrapure water. Water was ultrapurified by reverse osmosis with an Autwomatic Plus system (Water type II) followed by ion exchange with an 18.2 MΩ cm deionized Ultramatic Plus system (Wasserlab, Barbatáin, Navarra, Spain). All sample handling and preparation of solutions were carried out using acid cleaned material, wearing polyethylene gloves and under laminar flow cabinet Cruisair 870-FL (Cruma, Saint Boi de Llobregat, Barcelona, Spain).

### 3.4. Exploratory Analysis of Chemical/Elements Compositional Data

To explore data patterns and assess the expected variations in chemical composition among samples collected in 2025 in different sites and their relationships with sediment characteristics, both Pearson correlation analysis and Principal Component Analysis (PCA) were applied. PCA was carried out on standardized data referred to both 2023 and 2025 campaigns. The seasonal influence was also studied by comparison of the results from those obtained in preliminary research of the authors [4]. The samplings of 2023 for comparison were in July (labeled sampling 2nd) and September (labeled sampling 3rd)). Therefore, the 2025 sampling was referred to as 4th one. Pearson correlation coefficients were calculated using MATLAB R2024b [29] to assess the linear relationships between TE concentrations, grain size fractions and mineralogical components. PCA was conducted using the PLS_Toolbox 9.5 (2024) [30]. The analysis was performed with data following logarithmic transformation and autoscaling. Pearson correlation coefficients were calculated to assess linear relationships among metal concentrations, grain-size fractions and mineralogical components. For interpretative purposes we consider correlations qualitatively as follows: weak (|r| < 0.4), moderate (0.4 ≤ |r| < 0.7), and strong (|r| ≥ 0.7).

### 3.5. Pollution and Risk Assessment Indices

Several indices were obtained to evaluate the quality of the sediment and to assess the degree of contamination by comparing with background levels of metal, following the methodologies outlined by [21,31]. Background concentrations were derived from regional studies of central Adriatic sediments [9,21]. Aluminum was selected as reference element in EF due to its conservative lithogenic behavior and minimal anthropogenic input. This element is commonly used for normalization as it is one of the most abundant elements on Earth and generally presents no significant contamination concerns. Studies of sediment from the Gulf of Trieste demonstrated a strong correlation between Al and the fine fraction (silt + clay), making it an ideal proxy for this purpose [14].

Contamination factor (*Cf*) is an index used to evaluate the contamination of a specific substance in a basin.

*Cf = Cs/Cb* (1)

where *Cs* is the concentration of the substance in sediment, and *Cb* is the background values for the same element. The Contamination degree (*C_d_*) is the sum of all *Cf* for various TEs and the Modified Contamination degree (*mC_d_*) is an overall average value for a range of pollutants (the number of TEs considered (*n*)) (Equation (2)). The Enrichment factor (*EF*) evaluates the metal contamination and enrichment degree, and it normalizes the trace element content with respect to a sample reference metal, such as Al (Equation (3))

(2) $$mC_{d}=\frac{C_{d}}{n}=\frac{\sum_{1}^{n}Cf}{n}$$

(3) $$EF=\frac{{\left(\frac{M}{Al}\right)}_{sediment}}{{\left(\frac{M}{Al}\right)}_{background}}$$

The Geoaccumulation index (*Igeo*) gives an assessment of TEs contamination of sediments with respect to the background natural levels (Equation (4)). The Pollution Load Index (*PLI*) evaluate the contamination status of sediments to TEs (Equation (5)).

(4) $$Igeo={log}_{2}\frac{Cs}{1.5\times Cb}$$

(5) $$PLI=\sqrt[{n}]{Cf1\times Cf2\times \ldots Cfn}$$

The Potential Ecological Risk Index (*PERI*) is the sum of Potential Ecological Risk factor, which can be calculated using the toxicity response index of elements proposed by [3] for selected elements Cu, Cr, Cu, Ni, Pb and Zn. The formula for the calculation of the potential ecological risk factor ($E_{f}^{i}$) of single metal pollution is:

(6) $$PERI=\sum E_{f}^{i}=\sum C_{f}^{i}\times T_{f}^{i}$$

where $T_{f}^{i}$ is toxic response factor for a given element (Cd = 30, Cr = 2, Cu = 5, Ni = 5, Pb = 5, Zn = 1) and $C_{f}^{i}$ is the Contamination factor for each metal. The Availability Index *AI_tot_* is calculated based on the difference between the metal content in the residual fraction (*C_residue_*) obtained through the BCR extraction and the pseudo-total metal content (*C_tot_*) (Equation (7)):

(7) $${AI}_{tot}=\sqrt[{n}]{{AI}_{m1}\times {AI}_{m2}\times \ldots {AI}_{mn}}=\sqrt[{n}]{{\left(\frac{C_{tot}-C_{residue}}{C_{residue}}\right)}_{m1}\times {\left(\frac{C_{tot}-C_{residue}}{C_{residue}}\right)}_{m2}\times ...{\left(\frac{C_{tot}-C_{residue}}{C_{residue}}\right)}_{mn}}$$

## 4. Conclusions

This study provides a comprehensive assessment of trace metal contamination and ecological risk in marine sediments of the Tremiti Islands. The results reveal spatial variability of metal concentrations, with Cala Spido and Grotta del Sale showing the highest levels of contamination indices, particularly for As, Cd, Cr, Cu, Mn, Pb and Ti. Pollution indices (*mC_d_* and *PLI*) generally indicate low to moderate contamination. While other metals appear to be mainly of lithogenic origin and bound to residual fractions, a notable portion of potentially available metals was detected, especially in fine-grained, quartz-rich sediments.

Importantly, a temporal decrease in Cd concentrations and associated risk indicators (*mC_d_* and *PERI*) were observed in the most recent sampling (January 2025) at several sites, suggesting a potential reduction in contaminant inputs due to the winter season or natural attenuation processes.

Multivariate analysis confirmed the influence of both natural and anthropogenic sources, as well as the key role of sediment granulometry in metal distribution. Overall, the integration of geochemical, granulometric and statistical data supports a nuanced understanding of metal behavior in coastal marine environments and highlights the importance of long-term monitoring to capture both spatial patterns and temporal dynamics in protected areas.

## Figures and Tables

**Table 1:** Mean values of pseudo-total element concentration (mg/kg dry weight), standard deviations and percentage of residual fraction (F4) after the BCR extraction (nd: not detected (<LOD)). Data from 2025 campaign.

Site	Value	Ag	Al	As	Ba	Be	Cd	Co	Cr	Cu	Fe	Mn	Mo	Ni	Pb	Sb	Se	Sn	Ti	Tl	V	Zn
**P**	**Mean**	nd	458	2.26	6.44	nd	0.30	0.67	4.39	1.84	487	63.6	nd	2.04	3.08	nd	0.92	nd	9.81	nd	4.14	9.72
	**SD**	-	31	0.29	0.42	-	0.01	0.07	0.23	0.82	23	9.2	-	0.19	0.09	-	0.08	-	0.15		0.04	2.60
	**%F4**	nd	9	nd	7	nd	nd	nd	12	nd	9	0.84	nd	3	24	nd	nd	nd	33	nd	5	45
**CM**	**Mean**	nd	2.13 × 10^3^	2.90	28.1	0.13	nd	1.54	8.73	6.1	1.76 × 10^3^	95.8	0.14	5.22	11.5	nd	1.35	nd	47.5	nd	8.54	23.3
	**SD**	-	111	0.16	6.9	0.02	-	0.34	0.50	5.0	62	9.2	0.01	0.02	2.1	-	0.16	-	5.3		0.04	10.4
	**%F4**	nd	61	45	31	55	nd	35	49	9	96	13	51	47	20	nd	8	-	78	nd	45	42
**CS**	**Mean**	nd	2.51 × 10^3^	4.62	24.6	0.15	nd	1.68	10.1	2.73	2.11 × 10^3^	108	0.16	6.64	6.15	nd	1.15	0.28	57.7	nd	8.89	29.7
	**SD**	-	29	0.03	8.8	0.01	-	0.08	0.2	0.35	66	2	0.01	0.45	0.06	-	0.12	0.08	2.1		0.06	8.0
	**%F4**	nd	47	32	27	43	nd	42	48	17	63	5	57	49	28	nd	8	-	48	nd	40	50
**GS**	**Mean**	nd	5.21 × 10^3^	3.34	31.4	0.24	0.19	1.72	11.9	3.92	3.05 × 10^3^	110	0.42	6.31	6.80	0.77	1.30	0.40	81.8	nd	12.9	24.4
	**SD**	-	202	0.08	0.4	0.01	0.01	0.04	0.4	0.12	242	8	0.04	0.28	0.23	0.04	0.01	0.08	0.8	-	0.76	11.1
	**%F4**	nd	73	36	45	65	nd	59	74	26	88	14	11	72	64	102	29	-	74	nd	60	91

**Table 2:** Pollution and risk assessment indices (*C_d_*, *mC_d_*, *PLI*, *PERI* and *AI_tot_* are calculated on all the metals analyzed).

Label	1. P				2. CM				3. CS				4. GS			
	*Cf*	*Igeo*	*EF*	Ei	*Cf*	*Igeo*	*EF*	Ei	*Cf*	*Igeo*	*EF*	Ei	*Cf*	*Igeo*	*EF*	Ei
As	0.2	−2.7	34,0		0.3	−2.4	43.8		0.5	−1.7	69.6		0.3	−2.2	50.3	
Cd	0.7	−1	110	22.1	0.6	−1.4	86.1	17.1	0.7	−1.2	101.3	20.2	0.5	−1.7	70.7	14.1
Cr	0	−5.5	5.2	0.2	0.1	−4.5	10.3	0.3	0.1	−4.3	11.8	0.4	0.1	−4	14	0.5
Cu	0	−5	6.9	0.2	0.2	−3.3	23	0.8	0.1	−4.5	10.3	0.3	0.1	−3.9	14.8	0.5
Mn	0.1	−4.2	12.4		0.1	−3.6	18.6		0.1	−3.4	21.1		0.1	−3.4	21.5	
Pb	0.2	−3.1	25.8	0.9	0.6	−1.2	96.3	3.2	0.3	−2.1	51.5	1.7	0.4	−2	56.9	1.9
Ti	32.7	4.4	4.93 × 10^3^		158	6.7	2.39 × 10^4^		192	7	2.90 × 10^4^		273	7.5	4.11 × 10^4^	
Zn	0.2	−2.9	30.5	0.2	0.5	−1.6	73.1	0.5	0.6	−1.3	93.2	0.6	0.5	−1.6	76.6	0.5
	***C_d_***	34.3	***PERI***	23.5	***C_d_***	161	***PERI***	21.9	***C_d_***	195	***PERI***	23.2	***C_d_***	274	***PERI***	17.4
	***mC_d_***	2.6	***AI_tot_***	9.3	***mC_d_***	12.4	***AI_tot_***	1.38	***mC_d_***	15	***AI_tot_***	1.86	***mC_d_***	22.9	***AI_tot_***	0.78
	***PLI***	0.1			***PLI***	0.3			***PLI***	0.3			***PLI***	0.4		

**Table 3:** Localization of sampling sites and sampling conditions (January 2025).

	Site	Long °E (gg pp ss.dd)	Lat °N (gg pp ss.dd)	Depth (m)	Temperature (°C)
1.	Pagliai (P)	15°29′46.65′′ E	42°06′43.13′′ N	10.3	16.7
2.	Cala Spido (CS)	15°29′45.89′′ E	42°06′59.65′′ N	10.0	16.9
3.	Cala Matano (CM)	15°29′38.90′′ E	42°06′50.24′′ N	15.0	16.5
4.	Grotta del sale (GS)	15°29′29.11′′ E	42°06′31.94′′ N	19.0	16.7

## Data Availability

Data are included in this article/referenced in this article. Other data will be available on request.

## Appendix A

**Table A1:** Validation of TEs analysis using BCR 701 reference material (lake sediment) for aqua regia extractable contents.

TE	$\bar{x}$ (mg/kg)	Indicative Values BCR 701 (mg/kg)	Recovery (%)
Cd	12.1 ± 1.2	11.7 ± 1.0	103
Cu	239 ± 17	275 ± 13	88
Cr	265 ± 33	272 ± 20	97
Pb	134 ± 12	143 ± 6	94
Ni	98 ± 9	103 ± 4	96
Zn	390 ± 24	454 ± 19	86

**Table A2:** Validation of TEs analysis using BCR 701 reference material (lake sediment) for F4 fraction.

TE	$\bar{x}$ (mg/kg)	Indicative Values BCR 701 (mg/kg)	Recovery (%)
Cd	0.11 ± 0.06	0.13 ± 0.08	85
Cu	38.1 ± 3.3	38.5 ± 11.2	99
Cr	54.5 ± 1.7	62.5 ± 7.4	87
Pb	12.2 ± 1.6	11.0 ± 5.2	111
Ni	37.8 ± 2.5	41.4 ± 4.0	91
Zn	81 ± 12	95 ± 13	85
